# From ketogenic metabolism to targeted therapeutics: current advances in β-hydroxybutyrylation

**DOI:** 10.3389/fimmu.2025.1690224

**Published:** 2026-01-15

**Authors:** Fangshuai Hu, Changming Liang, Xu Zhang, Lishuai Xu, Chengwei Wu, Senlin Wan, Jiawei Wang, Mengyun Wang, Dawei Zhang, Yongchun Liu, Zhenyuan Li, Xiaoming Wang, Xiaoxu Huang, Li Xu

**Affiliations:** 1 Department of Gastrointestinal Surgery, Yijishan Hospital of Wannan Medical College, Wuhu, Anhui, China; 2 Anhui Province Key Laboratory of Non-coding RNA Basic and Clinical Transformation, Wuhu, Anhui, China; 3 Department of Gastrointestinal Surgery, The Second Affiliated Hospital of Wannan Medical College, Wuhu, Anhui, China; 4 Department of Hepatobiliary Surgery, Yijishan Hospital of Wannan Medical College, Wuhu, Anhui, China; 5 Department of Gastrointestinal Surgery, Ren Ji Hospital, Shanghai Jiao Tong University School of Medicine, Shanghai, China

**Keywords:** gene regulation, immune regulation, predicted sites, tumour, β-hydroxybutyrylation

## Abstract

β-Hydroxybutyrylation (Kbhb) is a novel posttranslational modification (PTM) mediated by β-hydroxybutyrate (BHB). BHB, the core product of ketogenic metabolism, serves as its direct precursor and substrate. As a hub connecting energy metabolism and the epigenetic network, Kbhb exerts bidirectional regulatory effects on abnormal tumour metabolism, cardiovascular and cerebrovascular diseases, immune regulation, and other processes. Furthermore, Kbhb is not limited to histones; it is also widely present in nonhistones and influences various biological processes, such as protein stability, metabolic and energy homeostasis regulation, pathogen virulence regulation, transcriptional regulation, and signal transduction. This review summarizes the research progress in the field of Kbhb, including the inducers of Kbhb (ketogenic diet), prediction methods for modification sites (KbhbXG, pFunK, SLAM, iBhb-Lys), regulatory elements of modification (regulatory enzymes such as ENL and SIRT6, and protein substrates), mechanisms of action in cancer (e.g., mTOR signalling pathway, cGAS-STING signalling pathway), mechanisms of action in immune-related signalling pathways and immune-active components regulation, research progress on histone and nonhistone Kbhb (e.g., Bcl6, P53, STAT1, UvSlt2), and novel therapeutic strategies for diseases based on Kbhb modification (metabolic regulation and targeted therapy), providing new insights for targeted therapy for cancer and other diseases.

## 1 Introduction

Epigenetics refers to changes in gene expression levels caused by nongenetic sequence alterations. The main types of epigenetic “codes” include covalent DNA modifications, covalent protein modifications, and noncoding RNA (ncRNA) regulation (1). Proteins undergo various chemical modifications after translation, known as post-translational modifications (PTMs), which mainly include methylation, phosphorylation, acetylation, ubiquitination, SUMOylation, glycosylation, and ADP-ribosylation. As crucial regulators of protein function, PTMs play important regulatory roles in various biological processes and in the occurrence and development of diseases. For example, PTMs occurring on histones can affect gene expression by altering chromatin structure (2). Currently, a variety of new acylations, such as acetylation, ubiquitination, crotonylation, malonylation, succinylation, and glutarylation, have been discovered. These modifications are intimately involved in numerous biological processes, including metabolic regulation, epigenetic regulation, and signal transduction. Among them, lactylation has become a research hotspot in recent years due to its core role in metabolism (3). Ketogenic metabolism is an adaptive metabolic state activated by the human body to cope with energy crises under specific conditions, such as a very-low-carbohydrate diet (usually less than 50 grams per day), starvation, fasting, or prolonged exercise (4). Its core lies in the switch of energy sources: when glucose is scarce, the body mainly shifts to fat breakdown for energy supply. This process primarily occurs in the mitochondria of liver cells. The core products generated after fat breakdown are ketone bodies, which include mainly acetoacetate (AcAc), β-hydroxybutyrate (BHB), and acetone (ACE). Ketone bodies can serve as important alternative energy sources for vital tissues such as the brain and muscles, functions that hold crucial physiological significance for maintaining life activities during starvation and reflects the body’s survival adaptability. For a long time, researchers generally believed that the large amount of BHB produced during the ketogenic state can directly activate the expression of a series of protective genes, thereby exerting important functions such as antioxidation, anti-inflammation, and neuroprotection (5). It was not until 2016 that the research team led by Professor Yingming Zhao from the University of Chicago first reported a brand-new type of acylation—β-hydroxybutyrylation (also known as 3-hydroxybutyrylation, Kbhb) (6), which refreshed our understanding of this field. To date, histone Kbhb has been detected in yeast, Drosophila, mice, and human cells, and a total of 46 histone Kbhb sites have been identified both *in vivo* and *in vitro (*7). However, research on the Kbhb modification of nonhistones remains relatively limited. As it is a newly discovered modification type, exploring its enzyme system is particularly important for elucidating the modification mechanism and function of β-hydroxybutyrylation. Therefore, this review summarizes the inducers of Kbhb, prediction methods for modification sites, regulatory elements of modification, mechanisms of action in cancer, mechanisms of action in immune-related signalling pathways and immune-active components regulation, research progress on histone and nonhistone Kbhb, and novel therapeutic strategies for diseases based on Kbhb modification, aiming to provide new ideas for targeted therapy for cancer and other diseases.

## 2 An inducer of Kbhb: the ketogenic diet

**Figure 1:**
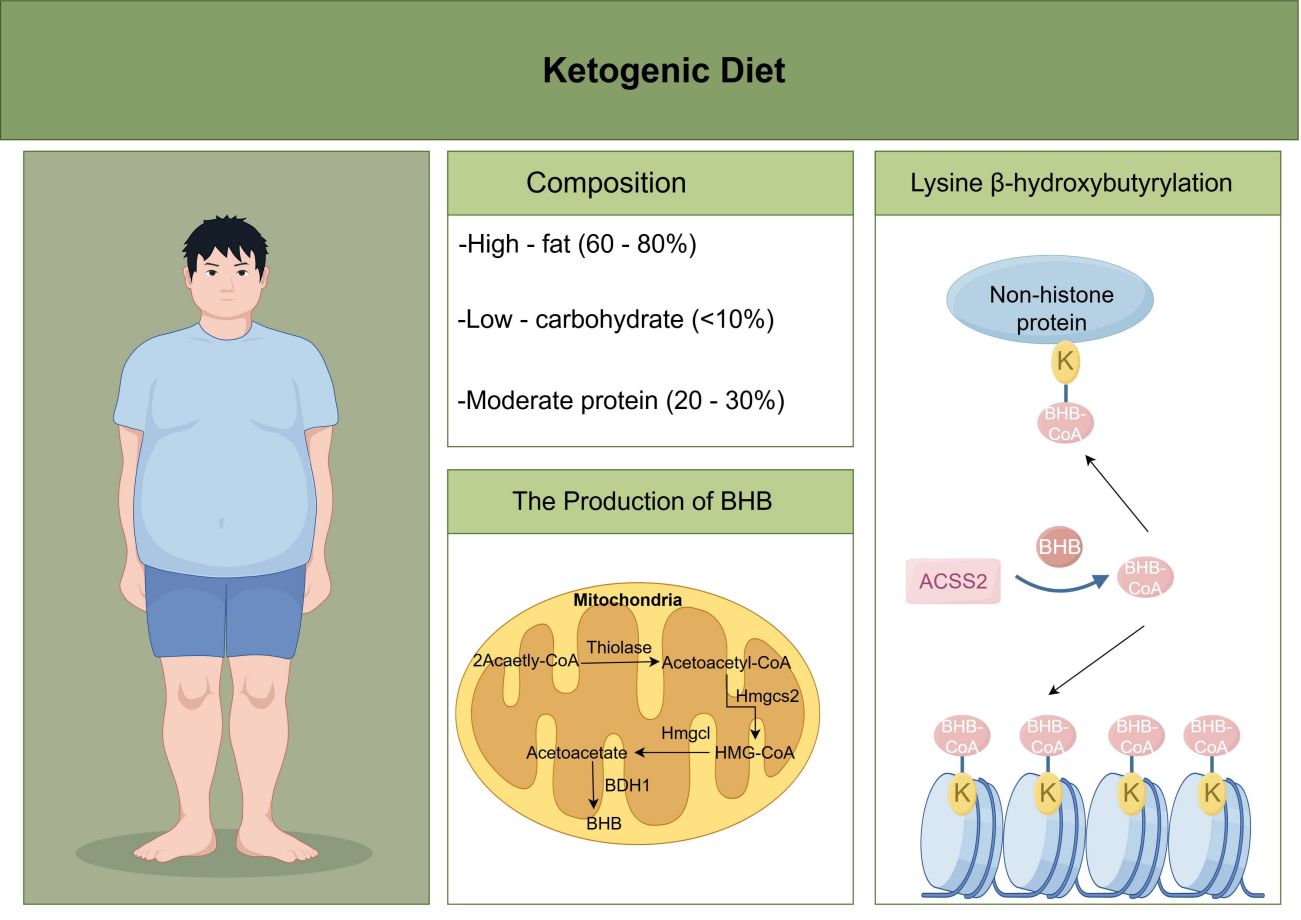
The dietary pattern of the ketogenic diet consists of high fat, very low carbohydrates, and moderate protein. Hepatic mitochondria can induce the production of BHB under the action of rate-limiting enzymes such as HMGCS2 and BDH1. Under the action of ACSS2, BHB combines with free CoA to form BHB-CoA, and further forms Kbhb modification on the lysine residues of proteins.

The ketogenic diet (KD) is a dietary pattern centred on high fat, very low carbohydrate, and moderate protein consumption. By simulating the body’s starvation state, it forces the energy metabolism mode to shift from glucose combustion for energy supply to fat breakdown for ketone body production. Studies have shown that KDs play roles in the treatment of neuropsychiatric disorders (8), metabolic and endocrine disorders (9), cardiovascular diseases (10), and tumours (11). Notably, in Kbhb, KDs are key inducers of this modification, and the BHB involved in protein Kbhb modification is derived from fatty acid β-oxidation during ketone body formation (12). Ketone bodies include mainly AcAc, BHB, and ACE, among which BHB has the highest content. In the ketogenic pathway, two enzymes—3-hydroxy-3-methylglutaryl-CoA synthase 2 (HMGCS2) and 3-hydroxybutyrate dehydrogenase 1 (BDH1)—play core roles: HMGCS2 catalyses the rate-limiting step of ketone body formation, converting acetyl-CoA into the ketone body precursor 3-hydroxy-3-methylglutaryl-CoA (HMG-CoA), and BDH1 is responsible for converting acetoacetate into BHB (13). During Kbhb modification, BHB binds to free coenzyme A (CoA), and the formed β-hydroxybutyryl-CoA (BHB-CoA) serves as a high-energy donor for Kbhb modification. Notably, Kbhb modifications differ among proteins: D-β-hydroxybutyrate (D-BHB) is one of the ketone bodies produced by the liver through ketogenic metabolism when glucose is scarce (e.g., fasting, intense exercise), and it is a common source of Kbhb modification (14); L-β-hydroxybutyrate (L-BHB) is not a naturally occurring free metabolite in the human body, and its activated form, L-BHB-CoA, is generated mainly transiently in the final stage of fatty acid β-oxidation (12). Under fasting or ketogenic conditions, the concentration of L-BHB-CoA increases, and L-BHB-CoA is involved in the Kbhb modification of mitochondrial proteins. The core difference between the two lies in the production pathway of their precursor metabolites: D-BHB is the main product of ketogenic metabolism, whereas L-BHB-CoA is an intermediate product of fatty acid β-oxidation, and L-BHB itself is not a freely existing metabolite. Previous studies have shown that butyrate and crotonate can be converted from their homologous short-chain fatty acids (SCFAs) into corresponding coenzyme A derivatives via acyl-CoA synthetase 2 (ACSS2) (15, 16). Recent research confirms that ACSS2 is also a key enzyme that regulates the conversion of BHB to BHB-CoA (17). In summary, under conditions such as fasting, exercise, calorie restriction, and a ketogenic diet, the liver can induce the production of BHB through fatty acid β-oxidation, which then binds to free CoA to form BHB-CoA, ultimately leading to Kbhb modification on the lysine residues of proteins (**Figure 1**).

## 3 Prediction methods for Kbhb modification sites

Kbhb is an important posttranslational modification closely related to various physiological processes, such as metabolic regulation, gene expression, and signal transduction. Accurate detection of Kbhb sites is a prerequisite and key for in-depth research on the molecular mechanism and biological function of β-hydroxybutyrylated proteins. Currently, the identification of Kbhb sites relies mainly on experimental methods such as mass spectrometry (18). However, these traditional experimental methods are both expensive and time-consuming. Therefore, the development of computational methods and prediction tools is highly important for the rapid identification of Kbhb sites. At present, computational methods for major PTMs such as phosphorylation (19) and acetylation (20) have been extensively explored (e.g., RF-phos and ProAcePred 2.0 tools), but specialized computational resources specific for β-hydroxybutyrylation modification remain relatively scarce. Therefore, the development of Kbhb site predictors based on machine learning will greatly promote the prediction and analysis of Kbhb sites, helping researchers quickly understand the functional significance related to this modification.

### 3.1 KbhbXG

KbhbXG is the first Kbhb modification site prediction tool designed based on machine learning technology proposed by Chen et al. (21). This tool identifies Kbhb modification sites based on protein sequence information through the XGBoost algorithm. The research team first constructed an optimized Kbhb modification dataset, taking it as the core of the benchmark dataset for Kbhb analysis. Subsequently, using the XGBoost algorithm with 10-fold cross-validation, they systematically evaluated and compared the prediction effects of four single-feature encoding schemes (Binary, BLOSUM62, CKSAAP, ZScale), as well as their pairwise combinations, three-way combinations, and fusion technologies. The results showed that the strategy of fusing multiple features could significantly improve the prediction performance of Kbhb site identification; among all feature types, the BLOSUM62 feature performed particularly prominently. This matrix can effectively capture short- and long-range correlation information between amino acids, and because of its significant statistical characteristics, high sensitivity to evolutionary relationships, and comprehensive characterization capabilities, it has become an ideal choice for Kbhb site prediction. Finally, the research team constructed the Kbhb PTM site prediction model KbhbXG based on the XGBoost algorithm and BLOSUM62 features. The dataset construction and evaluation of feature encoding schemes of this tool have led to important breakthroughs in the field of Kbhb site prediction, and it is expected to become an important analytical tool for screening candidate sites for experimental verification and facilitating the exploration of Kbhb functional mechanisms. Notably, limited by existing data, KbhbXG is currently applicable only to *Homo sapiens*; its prediction effects in other species still need further verification. In the future, the optimization of this tool can focus on exploring new feature encoding methods, introducing deep learning technologies, and integrating other bioinformatics tools and databases; at the same time, with the accumulation of Kbhb site data, timely updating of the training dataset is crucial for enhancing model stability and expanding the application scope. The continuous improvement and promotion of KbhbXG will provide strong support for the analysis of the regulatory mechanism and biological function of protein Kbhb.

### 3.2 pFunK

With the rapid development of proteomics technology, thousands or even tens of thousands of PTM sites can be identified in a single experiment. However, functional annotation of these sites remains challenging. Previously, to address this issue, Ochoa et al. (22) developed a machine learning algorithm called the “phosphosite functional score”, which trains a model using 2638 phosphosites with known functions to prioritize functional phosphorylation sites. However, the dataset relied on by this algorithm is small in scale, and direct application to conventional machine learning easily leads to high bias and overfitting of the model. To address this limitation, Qin et al. (23) developed a hierarchical learning framework, “predicting functionally important lysine modification sites (pFunK)”, which shows high accuracy in predicting functional Kbhb sites. The hierarchical learning logic of this framework is based on two core hypotheses: first, the transferable knowledge of upper-level tasks is mostly shared by lower-level tasks; second, all Kbhb sites possess the common characteristics of lysine modification sites, and Kbhb sites with functional importance further possess the specific characteristics of Kbhb sites. Based on the above hypotheses, pFunK performs accurate prediction of target modification sites through a three-step process: “pretraining a lysine modification model → transfer learning to construct a Kbhb model → fine-tuning a functional Kbhb model”. The research team conducted strict verification on the performance of pFunK: they selected 6 Kbhb sites predicted by pFunK for experiments and successfully verified 5 of them, indicating that this framework has good potential for predicting lysine modification sites, Kbhb sites, and functionally important Kbhb sites. In addition, pFunK can be extended to the study of other types of lysine acylation, such as lysine acetylation (Kac), lysine lactylation (Kla), and lysine crotonylation (Kcr), while maintaining high prediction accuracy.

### 3.3 SLAM

SLAM is a hybrid deep learning neural network proposed by Qin et al. (24). This model integrates structural and language model constraints and can realize universal and species-specific protein Kbhb site prediction. As an innovative hybrid deep learning framework, SLAM combines a protein language model with a graph neural network with structure-aware capabilities, significantly improving the prediction accuracy of Kbhb sites and showing obvious advantages in the task of computer-simulated Kbhb site prediction. From an application perspective, SLAM has broad practical value: it can aid in the discovery of new Kbhb sites closely related to human diseases, efficiently screen Kbhb sites with the potential for up- or downregulation, and provide important references for subsequent experimental verification; moreover, based on its structure-guided characteristics, SLAM can also be used to identify other key posttranslational modification types. Overall, SLAM provides a new perspective and method for detecting posttranslational modifications of great significance to protein structure and function and shows great application potential in the field of bioinformatics research.

### 3.4 iBhb-Lys

iBhb-Lys is another novel computational model proposed by Ju et al. for identifying Kbhb sites (25). Previous studies have shown that the development of efficient Kbhb site predictors focuses mainly on two core aspects: first, exploring effective feature extraction strategies to accurately characterize the characteristics of Kbhb sites; second, designing high-accuracy classification algorithms as the core support of the prediction model. On this basis, Ju et al. innovatively integrated four features—amino acid composition, amino acid factors, binary encoding, and k-spaced amino acid pair composition—to encode Kbhb sites. Although combined features cover more abundant information than single features do, they also cause problems such as a sharp increase in the feature space dimension and enhanced model complexity. To solve such problems, the research team constructed an autoencoder network (26), which can not only effectively reduce the dimension of the feature space but also independently learn abstract features from data to achieve efficient compression of the feature space. In addition, to reduce the interference of noise and outliers on the prediction results, the team proposed a fuzzy support vector machine algorithm. The unique feature of this algorithm is that it integrates the density information around samples into the fuzzy membership function and performs better than several common fuzzy SVM algorithms do. Through 10-fold cross-validation and independent testing, the model showed good performance and generalizability. The supporting iBhb-Lys web server can provide Kbhb site prediction and data download services for researchers. Notably, the technical framework of the iBhb-Lys model is expected to be extended to the prediction of other types of modification sites (**Table 1**).

**Table 1:** Prediction methods for Kbhb modification sites.

Tool name	Core technology	Advantages	Limitations	References
KbhbXG	XGBoost algorithm and BLOSUM62 characteristic coding	BLOSUM62 has strong statistical characteristics in capturing short/long-range correlations of amino acids. High model interpretability.	Only applicable to Homo sapiens. Generalization unvalidated due to reliance on limited data.	(21)
pFunK	Hierarchical learning framework	Efficient use of small sample data. Scalable to Kac/Kla/Kcr modifications.	Small training set size. Further validation needed for generalization across modification types.	(23)
SLAM	Protein language model and graph neural network	Balances species specificity and general prediction. Identifies disease-related sites and regulatory directions.	High computational resource requirements. Structural dependence may limit application to unstructured proteins.	(24)
iBhb- Lys	Autoencoder Network and Fuzzy Support Vector Machine Algorithm	Strong anti - noise ability, optimal performance in independent testing	Complex feature combination, parameters need optimization.	(25)

## 4 Regulatory elements of the Kbhb pathway

**Figure 2:**
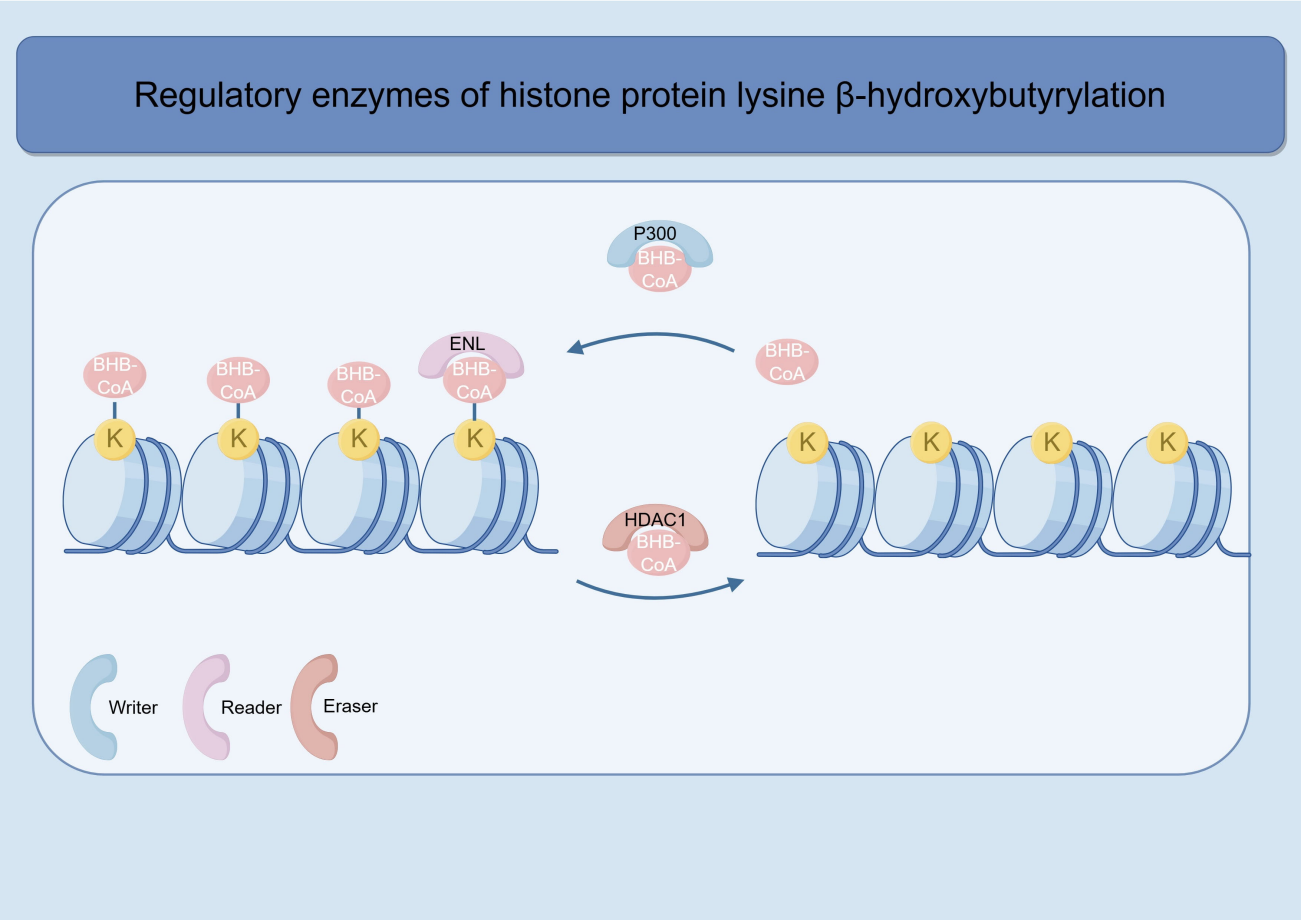
A dynamic process of β-Hydroxybutyrylation.

Over the past few decades, the regulatory mechanism and function of lysine acetylation (Kac) have been clarified through the identification of three core regulatory proteins: “writers”, “erasers”, and “readers”. Research on the identification and characterization of Kac “writers”, “erasers”, and “readers” and their interactions with cellular metabolism has made Kac among the most well-studied posttranslational modifications (PTMs) (27–29). Similarly, exploring the regulatory mechanism of Kbhb modification needs to start with these three types of proteins. The protein Kbhb is not only affected by key enzymes involved in ketone body metabolism and metabolic substrates themselves but also regulated by “writers”, “erasers”, and “readers”. “Writers” refer to enzymes that catalyse posttranslational modifications of proteins. According to recent studies, the acyltransferases p300 or CREB-binding protein (CBP) act as histone Kbhb “writers” to catalyse the addition of BHB to lysine, and this process can occur both *in vivo* and *in vitro (*30). Recent research has shown that the binding of histone deacetylase 1 (HDAC1) to HDAC3 can occur at active sites to catalyse the formation of Kbhb, and this process does not depend on the BHB-CoA intermediate (31). Wang et al. reported that ACSS2 couples with lysine acetyltransferase 7 (KAT7) to regulate histone H3K9bhb modification and promote transcription, among which KAT7 is the newly discovered “writer” enzyme for Kbhb modification (17). “Readers” refer to proteins or protein domains that can specifically recognize and bind to posttranslational modification sites of proteins. However, research on “readers” of histone Kbhb remains limited. Chen et al. (32)recently developed a self-assembled multivalent photoaffinity probe, which was used for the efficient and selective enrichment of histone Kcr “readers” and lysine 2-hydroxyisobutyrylation (Khib) “erasers” (33, 34). On this basis, the team further designed probes and combined them with quantitative proteomics methods to selectively analyse the binding substances of histone Kbhb. Using this method, they determined the interactors of H3K9bhb and identified ENL as a novel H3K9bhb “reader”. Enzymes that remove acylations are called “erasers”. Huang et al. reported that HDAC1 to HDAC3, as well as sirtuin 1 (SIRT1) and SIRT2, has de-Kbhb activity *in vitro*, but only HDAC1 and HDAC2 act as histone Kbhb deacetylases in cells (30). Sirtuins are a class of NAD^+^-dependent deacetylases, while HDAC1–3 are another typical class of zinc ion (Zn²^+^)-dependent deacetylases. Zhang et al. reported that SIRT3 exhibits class-selective histone de-β-hydroxybutyrylase activity, preferring H3K4, K9, K18, K23, K27, and H4K16 but not H4K5, K8, and K12, which distinguishes it from members of the zinc-dependent HDAC family (35). Notably, SIRT3 shows a greater preference for L-BHB (L-β-hydroxybutyrate) than D-BHB (D-β-hydroxybutyrate) (14), whereas HDAC3 has the opposite preference (36). Recent research revealed that SIRT6 is also an “eraser” for Kbhb modification; its rate of removing Kbhb is approximately 3 times slower than that of removing Kac but still significantly faster than the rate of removing other four-carbon acylations (37).Surprisingly, in plants, Oryza sativa Sirtuin 1 (OsSRT1), Oryza sativa Sirtuin 2 (OsSRT2), and Oryza sativa Histone Deacetylase 705 (OsHDA705) have been identified as “eraser” enzymes for histone Kbhb modification in rice (38). Meanwhile, the NAD^+^-dependent histone deacetylases Ustilaginoidea virens Sirtuin 2 (UvSirt2) and Ustilaginoidea virens Sirtuin 5 (UvSirt5) are the main enzymes that remove Kbhb, providing new insights into understanding the virulence regulation of phytopathogenic fungi (39). To date, only “erasers” have been confirmed to have the ability to stereospecifically recognize Kbhb isomers. To explore Kbhb-modified substrates, Huang et al. (30) conducted a study based on Kbhb pan-antibody-enriched protein modification proteomics and obtained the following key results: most Kbhb-modified proteins are localized in nuclear proteins, and only a small number of Kbhb-modified proteins are present in mitochondria; Kbhb-modified proteins are involved mainly in spliceosome, ribosome, RNA transport, and DNA repair-related pathways. In conclusion, Kbhb modification has a wide range of functions and is involved in various cellular processes, such as chromatin remodelling, transcriptional regulation, and DNA repair (**Figure 2**) (**Table 2**).

**Table 2:** Regulatory enzymes of Kbhb.

Regulatory enzynes	Regulatory proteins	References
Writers	p300/CBP、 HDAC1~3、KAT7	(17, 30, 31)
Readers	ENL	(32)
Erasers	HDAC1~3、SIRT1~3、SIRT6、UvSirt2 、 UvSirt5、OsSRT1、OsSRT2 、OsHDA705	(30, 35, 37–39)

## 5 Roles and mechanisms of Kbhb in cancer

### 5.1 mTOR signalling pathway

The mammalian target of rapamycin (mTOR) signalling pathway is an evolutionarily conserved multinode signal transduction pathway that integrates various extracellular signals, such as nutrient, energy, and growth factor signals. It is involved in biological processes, including gene transcription, protein translation, and ribosome synthesis, and plays crucial roles in cellular processes such as cell growth, apoptosis, autophagy, and metabolism. Notably, mTOR signalling is often abnormally activated during the occurrence and development of human cancers (40, 41). Previous studies have revealed an inherent mechanistic link between ketone metabolism and mTOR inhibition (42), and the latest research shows that Kbhb can effectively inhibit the activity of the mTOR signalling pathway, thereby suppressing cancer cell proliferation (23); the specific mechanism has been verified in various types of cancers. In liver cancer, reducing ketone body consumption or supplementing with BHB can induce Kbhb modification at lysine 108 of aldolase B (ALDOB) (ALDOB Lys108bhb). This modification impairs the enzymatic activity of ALDOB, hinders its binding to the substrate fructose-1,6-bisphosphate, and ultimately inhibits mTOR signal transduction and glycolysis, significantly reducing the proliferation rate of cancer cells. In addition, ALDOB Lys108bhb can downregulate mTOR signalling and glycolysis levels by reducing its binding ability to the substrate fructose-1,6-bisphosphate (FBP), thereby exerting significant inhibitory effects on the proliferation of various tumour cells, such as renal cancer, gastric cancer, and liver cancer cells.

### 5.2 cGAS-STING signalling pathway

The cyclic GMP-AMP synthase (cGAS)-stimulator of interferon gene (STING) signalling pathway is important for innate immune recognition in the body and plays key roles in antiviral defence, antibacterial infection, and tumour immune surveillance. Since the discovery of the cGAS enzyme in 2013 (43), research on this pathway has not only revolutionized people’s understanding of the “DNA sensing” mechanism but also become a core hub connecting research in multiple fields, such as infection, cancer, autoimmune diseases, and ageing. In the field of cancer, the cGAS-STING signalling pathway plays a dual role: on the one hand, activating this pathway can increase the body’s antitumour immune capacity and effectively inhibit tumour cell growth; on the other hand, tumour cells adopt various strategies to achieve immune escape, such as reducing cGAS expression or interfering with the normal transport of STING protein, thereby accelerating tumour progression (44). Recent research has shown that in enzalutamide-resistant castration-resistant prostate cancer (CRPC), ketogenesis-induced Kbhb activity inhibits the activation of the cGAS-STING signalling pathway (45). Compared with that in castration-sensitive prostate cancer, the expression of ketogenic pathway-related enzymes is significantly upregulated in CRPC. These changes lead to the formation of an “immune desert” in the tumour microenvironment, thereby triggering resistance to immunotherapy. In-depth mechanistic studies have revealed that upregulation of the ketogenic pathway promotes the massive accumulation of BHB in cells and that this BHB can promote Kbhb modification of the deubiquitinating enzyme OTUD7B. This modification inhibits the degradation of substrates by the anaphase-promoting complex/cyclosome (APC/C) in cells, reduces the accumulation of double-stranded DNA in the cytoplasm, and ultimately prevents the effective activation of the cGAS-STING signalling pathway, significantly reducing the expression level of interferons and impairing the body’s immune response capacity.

### 5.3 Regulation of gene expression

**Figure 3:**
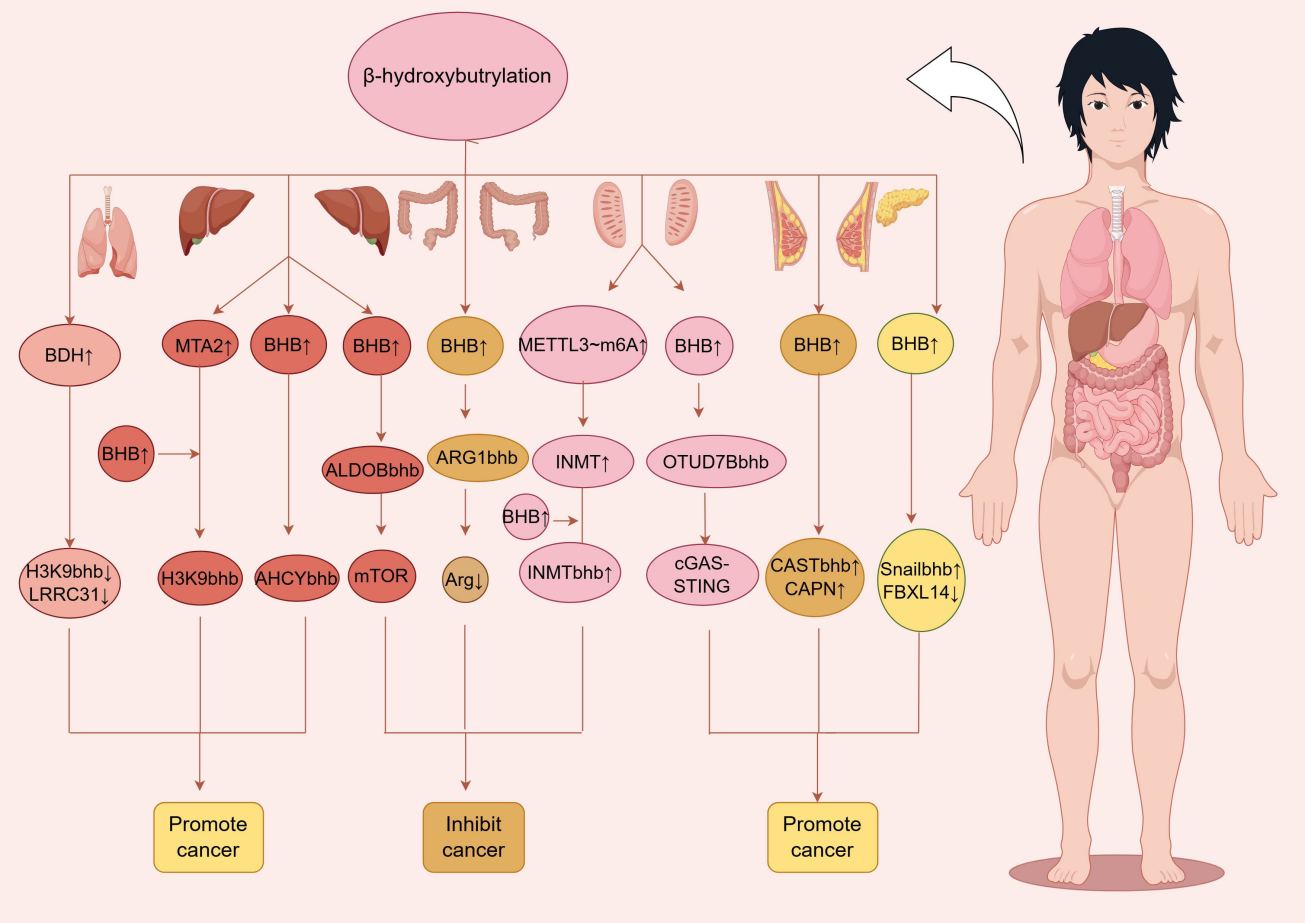
The roles and mechanisms of Kbhb in cancers,including promoting and inhibiting cancer progression.

Metastasis-associated protein 2 (MTA2) is associated with the invasive malignant phenotype of various cancers, but its specific molecular mechanism is still under investigation (46). Recent research has shown that MTA2 is highly expressed in hepatocellular carcinoma (HCC) cells and is localized mainly in the nucleus; moreover, MTA2 can specifically bind to HDAC2 and Chromodomain Helicase DNA-Binding Protein 4 (CHD4) and inhibit the transcription of the BDH1 gene through the R-loop structure. This regulation leads to the massive accumulation of BHB in cells, significantly increasing the level of Kbhb modification at the H3K9 site, thereby promoting the formation and progression of liver cancer. In addition, five key genes—JMJD6, GREB3, GTPBP4, NPM1, and TIMM23—have been confirmed to be closely associated with poor prognosis in patients with HCC, and the expression and functional activity of these genes can be dynamically regulated by the “MTA2–Rloop–BDH1–Kbhb” regulatory axis (47). In addition, studies have shown that Kbhb modification at the K389 and K405 sites of S-adenosyl-L-homocysteine hydrolase (AHCY) can increase its activity and promote the proliferation of HCC (18). Among breast cancers, triple-negative breast cancer (TNBC) has the worst prognosis and is usually associated with a high metastatic tendency (48, 49). Jiang et al. (50) reported that upregulated ketogenesis promotes TNBC metastasis by enhancing the biosynthesis of endogenous BHB. The increased biosynthesis of endogenous BHB further promotes β-hydroxybutyrylation modification of calpastatin (CAST) at the K43 site. This modification weakens the inhibitory effect of CAST on its endogenous target protein calpain (CAPN); the upregulated CAPN then promotes the invasion and metastasis of TNBC cells by increasing FAK phosphorylation and the epithelial–mesenchymal transition (EMT) process. Prostate cancer (PCa) is the second most common malignant tumour in men and the fifth leading cause of cancer-related deaths in both developed and developing countries, accounting for a large proportion of the global health burden (51). Recent research has shown that INMT is an oncogene in PCa that is highly expressed in PCa cells through the METTL3–N6-methyladenosine (METTL3-m6A) pathway and can promote the stem cell-like properties of these cells. Previous studies have shown that INMT may undergo Kbhb modification (18); the transcription factor SOX2 can endow cells with stem cell-like characteristics and promote a malignant, invasive phenotype during PCa development (52), and INMT can increase the activity of the SOX2 promoter. Further research revealed that BHB can inhibit the expression of INMT through Kbhb modification of INMT, thereby suppressing the malignant phenotype of PCa (53). Lung adenocarcinoma (LUAD) is the most common form of lung cancer and has a poor prognosis (54). A study by Huang et al. (55) confirmed that BDH1 is not only significantly overexpressed in LUAD but also regulates the overall level of intracellular Kbhb modification, promoting the malignant phenotype of cancer. Leucine-rich repeat-containing protein 31 (LRRC31) is a downstream target gene regulated by BDH1, and overexpression of LRRC31 significantly inhibited BDH1 overexpression-induced cell proliferation, stem cell-like characteristics, migration, and invasion. In-depth mechanistic analysis revealed that BDH1 expression in cells inhibits the transcription of LRRC31 by blocking the accumulation of H3K9bhb at the transcription start site of LRRC31, thereby promoting lung cancer progression (**Figure 3**).

## 6 Role of Kbhb in the regulation of immune-related signalling pathways and immune-active components

The immune system protects organisms from infection through a variety of defence mechanisms (56). Many studies have shown that an increasing number of histone or nonhistone acylations can effectively regulate various functions, processes, and diseases of the immune system, as well as related signalling pathways. “Immunometabolism”, as an emerging concept, describes the metabolic reprogramming of immune cells during activation and differentiation (57). Various PTMs that alter phenotypes and regulate immunity and inflammation under different conditions have been discovered in macrophages and T cells. Protein PTMs are becoming key means of regulating immunity through intracellular metabolites, and adverse changes in their levels can lead to immune disorders (58). As an emerging PTM, Kbhb also plays significant roles in immune-related signalling pathways and immune-active components regulation.

### 6.1 IFN-I signalling pathway

Understanding and slowing the ageing process is a major challenge. Ageing leads to declines in immune function, such as lymphopenia and abnormal macrophage function (59, 60). However, how PTMs participate in this process remains unclear. Recent research has shown that signal transducer and activator of transcription 1 (STAT1) undergoes two novel modifications: Kbhb modification and O-GlcNAc modification. These two modifications regulate the type I interferon (IFN-I) signalling pathway through antagonistic effects, thereby affecting the antiviral ability of elderly individuals (61). The team reported that ageing promotes Kbhb modification of STAT1 at lysine 592, which inhibits the interaction between STAT1 and IFN-I receptor 2 (IFNAR2) and hinders STAT1 tyrosine phosphorylation and the expression of downstream ISG genes, thereby impairing IFN-I-mediated antiviral defence. O-GlcNAc modification of STAT1 at threonine 699 prevents CBP-induced Kbhb modification of STAT1. However, the level of O-GlcNAc modification decreases in ageing tissues, which instead exacerbates the inhibitory effect of Kbhb. This study not only reveals the importance of the switch between STAT1 Kbhb and O-GlcNAc modifications in regulating IFN-I antiviral immunity during ageing but also provides two intervention strategies—hydroxycamptothecin and fructose—laying a theoretical foundation and translational path for improving the antiviral immunity of elderly individuals.

### 6.2 mTORC1 signalling pathway

Recent research has shown that BHB can regulate gene expression through its unique histone Kbhb modification, thereby exerting a renoprotective effect (62). Interestingly, inhibition of the mammalian target of rapamycin complex 1 (mTORC1) signalling pathway is a key downstream effect of BHB in achieving renal protection. The inhibition mechanism is not a direct effect of BHB but is indirectly achieved through its epigenetic effects to reshape cellular metabolism.High levels of BHB induce H3K9bhb modification in the kidneys of Dahl salt-sensitive rats, activating the transcription of lipid metabolism-related genes (such as Hmgcs2 and acetyl-CoA acetyltransferase 1B (Acaa1b)). The upregulated expression of these genes promotes intracellular fatty acid oxidation and is accompanied by peroxisome proliferation. This metabolic reprogramming puts cells in a state similar to energy restriction, thereby inhibiting the activity of the mTORC1 signalling pathway, ultimately leading to increased cellular autophagy, reduced protein synthesis, and weakened inflammatory responses.

### 6.3 NF-κB signalling pathway

Diabetic cardiomyopathy (DbCM) is a common cardiovascular complication in diabetic patients, and the expression of BDH1 is downregulated in diabetic hearts. Recent research has shown that BDH1 deficiency in mice leads to BHB accumulation, which in turn increases H3K9bhb modification, thereby promoting the transcription of lipocalin 2 (LCN2). Upregulated LCN2 can significantly promote the phosphorylation of nuclear factor κB (NF-κB) and its nuclear translocation by enhancing the interaction between ribosomal protein S3 (RPS3) and NF-κB. Activated NF-κB in the nucleus can further bind to the promoter regions of downstream inflammation- and apoptosis-related target genes, ultimately activating the inflammatory response and apoptosis pathways in cardiomyocytes (63).

### 6.4 Immune antibodies

In the context of the global pandemic of severe acute respiratory syndrome coronavirus 2 (SARS-CoV-2), effective anti-SARS-CoV-2 drugs are urgently needed. One of the most effective ways to combat SARS-CoV-2 infection is the use of antibody-based drugs. A study by Li et al. revealed that the SARS-CoV-2-neutralizing antibody B38 produced by 293T cells undergoes Kbhb modification *in vivo*, and this modification can increase the stability of the antibody, providing strong support for improving the therapeutic effect of SARS-CoV-2 antibodies (64). Specifically, Kbhb modification occurs on the positively charged lysine residues in the heavy and light chains of the B38 antibody. This discovery provides a potentially feasible approach for extending the protective effect of anti-SARS-CoV-2 antibodies.

### 6.5 Tmem cells

Previous studies have shown that the long-term survival of CD8^+^ memory T cells (Tmem) depends on fatty acid oxidation for energy, but this process also produces reactive oxygen species (ROS), which threaten cell survival (65, 66). However, Zhang et al. reported that the glycogen metabolism program guided by cytoplasmic phosphoenolpyruvate carboxykinase (Pck1) can promote Tmem cell formation by maintaining redox balance (67), and its specific regulatory mechanism is closely related to BHB-mediated Kbhb modification. Studies have confirmed that the ketogenesis product BHB can upregulate the expression of Foxo1 and Ppargc1a (which encodes PGC-1α) through H3K9bhb modification of their promoter regions. Subsequently, forkhead box protein O1 (FoxO1) and PGC-1α synergistically promote the expression of Pck1; Pck1 further directs carbon flow to the gluconeogenesis–glycogenolysis cycle and the pentose phosphate pathway, generating NADPH and glutathione (GSH). These two substances can effectively scavenge ROS, shifting the cellular metabolic program to antioxidant defence and ultimately supporting the long-term survival and functional memory formation of CD8^+^Tmem cells. In addition, during cellular energy metabolism, amino acid deamination produces ammonia as a byproduct, which is cytotoxic and can impair the long-term survival ability of cells. Although previous studies have shown that Tmem cells can effectively scavenge ROS, their ammonia detoxification mechanism has long been unclear. Recent research has shown that Tmem cells use the urea and citrulline cycles to scavenge ammonia, making memory development possible (68); BHB also plays a key regulatory role in this process. Studies by Tang et al. revealed that the ketogenesis product BHB upregulates the expression of the carbamoyl phosphate synthase 1 (Cps1) gene through P300-mediated H3K9bhb modification. High expression of CPS1 initiates the urea cycle, converting ammonia into urea; the arginine produced during the cycle is processed through two parallel pathways: In the urea cycle, arginine enters mitochondria through the transporter SLC25A29 and is catalysed by arginase 2 (ARG2) to generate urea; the urea is then excreted through SLC14A1. In the citrulline cycle, arginine is catalysed by nitric oxide synthase (NOS) in the cytoplasm to generate NO and citrulline, and citrulline reenters the cycle for metabolism. These two pathways efficiently scavenge ammonia to prevent toxicity, ensuring the long-term survival and functional persistence of CD8^+^Tmem cells, thereby enhancing the body’s immune memory.

### 6.6 CD4^+^T cells

Recent research revealed that patients with severe COVID-19 have defects in ketone body production, with significantly reduced serum BHB levels. Moreover, the function of CD4^+^T cells in the patient’s bronchoalveolar lavage fluid (BALF) and blood is exhausted, the production of key cytokines such as interferon-γ (IFN-γ) is reduced, and the expression of fibrosis-related genes is upregulated (69). This study revealed the core mechanism of BHB activity: as an alternative carbon source, BHB directly promotes the mitochondrial metabolism of T cells, shifting exhausted T cells from glycolytic metabolism to more efficient oxidative phosphorylation (OXPHOS) metabolism. Moreover, studies have confirmed that a ketogenic diet or direct exogenous supplementation of BHB can reprogram the metabolism of lung CD4^+^T cells; restore OXPHOS; promote IFN-γ production; reduce Programmed Death 1 (PD-1) expression; alleviate lung injury, fibrosis, and collagen deposition; and significantly reduce mortality. Although the specific proteins that undergo Kbhb modification have not been identified, this study proposes an innovative therapeutic strategy for regulating immune responses through a ketogenic diet or BHB supplementation. In addition, BHB-mediated Kbhb modification can also regulate the differentiation direction of CD4^+^T cells by affecting the function of transcription factors. After B-cell lymphoma 6 (Bcl6) undergoes Kbhb modification, it can significantly inhibit the differentiation of naive CD4^+^T cells into Tfh cells, leading to a reduction in the number of Tfh cells and a simultaneous decrease in the secretion level of the cytokine IL-21. This discovery provides a new perspective and a theoretical basis for exploring the regulatory mechanism of Tfh cell differentiation and the *in vivo* immune regulation process.

### 6.7 Macrophages

**Figure 4:**
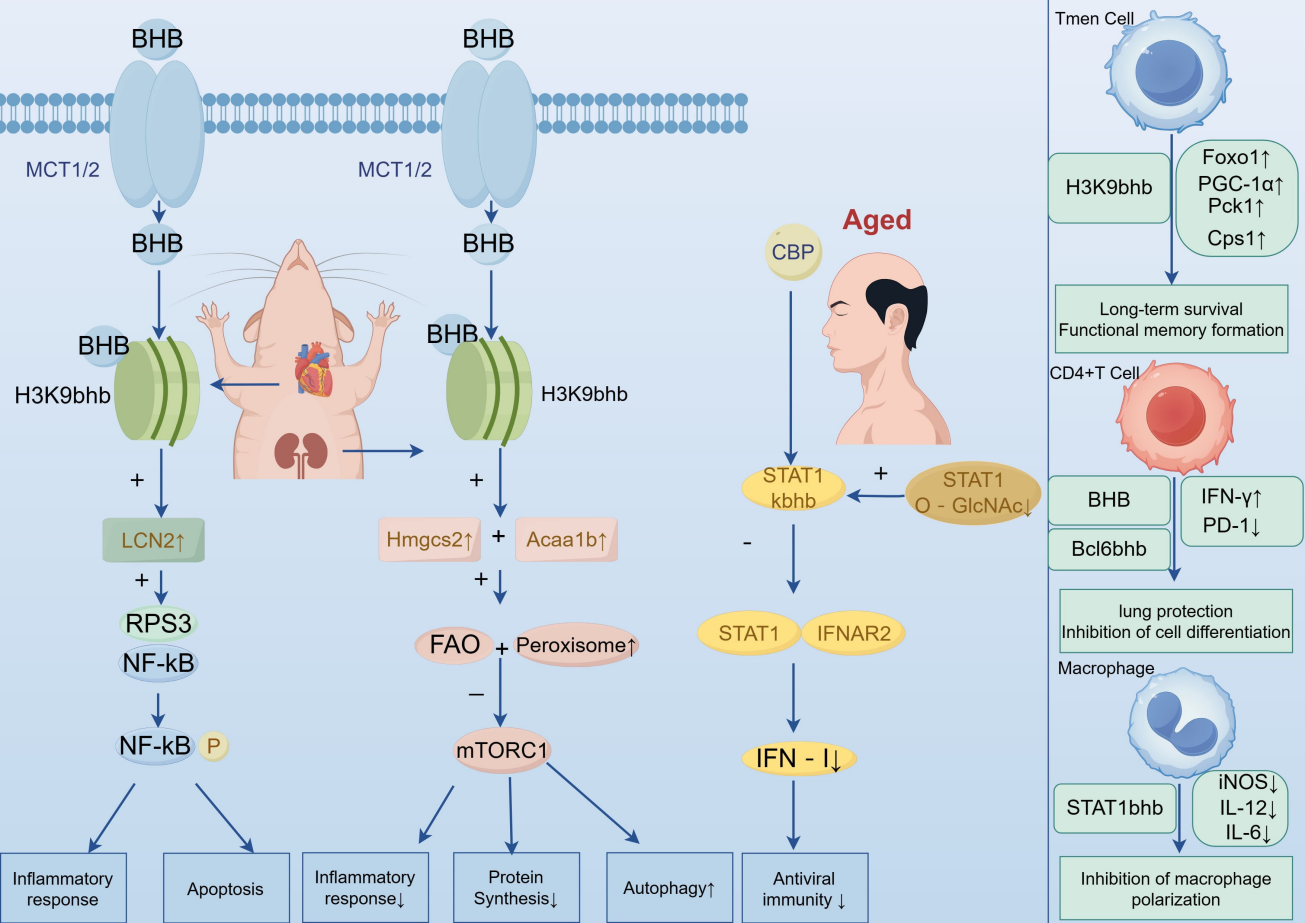
Role of Kbhb in the regulation of immune-related signalling pathways and immune-active components.

Macrophages are pleiotropic immune cells that are necessary for maintaining tissue homeostasis and regulating immune responses. Previous studies have shown that macrophages can differentiate into two different phenotypes in different immune microenvironments and play roles in immune regulation and inflammatory responses (70). Recent research has shown that BDH1 deficiency in macrophages leads to BHB accumulation. The accumulated BHB induces Kbhb modification of the STAT1 protein at its K679 site. This Kbhb modification inhibits the phosphorylation of the STAT1 protein at the Y701 site under lipopolysaccharide (LPS) stimulation, thereby inhibiting the transcriptional activation function of STAT1. Ultimately, the expression levels of key genes involved in M1 macrophage polarization (such as inducible nitric oxide synthase gene, IL-12, and IL-6) are downregulated, thereby inhibiting the polarization of macrophages to the proinflammatory M1 phenotype (71). This discovery provides a new molecular mechanism for the anti-inflammatory effect of BHB and reveals the important role of Kbhb modification in immunometabolic regulation (**Figure 4**).

## 7 Research progress on histone Kbhb: gene transcription activation

**Figure 5:**
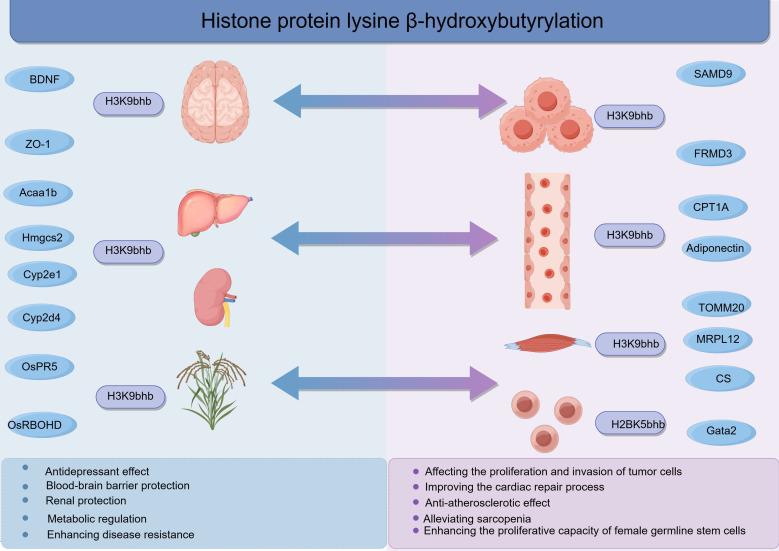
Research progress on histone Kbhb.

As a novel epigenetic mark, histone Kbhb is closely associated with the activation of gene transcription. Previous studies have demonstrated that it promotes transcriptional activation by targeting and becoming enriched in the promoter regions of genes, thereby regulating biological activities (6). Chromatin accessibility serves as the master switch for gene regulation; however, research on the relationship between histone Kbhb and chromatin accessibility is lacking. Studies have shown that H3K56bhb can precisely regulate gene expression by influencing the three-dimensional structure of chromatin, interacting with specific proteins (such as BRD4), and becoming enriched in key regions for gene regulation (such as superenhancers) (72). Scholars, including Xu, have reported that exogenous supplementation with BHB can induce H3K9bhb in rice; surprisingly, it also significantly enhances chromatin accessibility. The authors proposed that H3K9bhb may neutralize the positive charge of histones, weaken the interaction between histones and DNA, and thus promote chromatin opening (38). In summary, histone Kbhb can target and become enriched in gene promoter regions while increasing chromatin accessibility and can regulate biological activities by activating gene transcription. Although it is known that histone Kbhb can promote gene transcription, research on histone Kbhb has focused mainly on H3K9bhb. Recent research has shown that BHB has significant hypotensive and renoprotective effects on Dahl salt-sensitive rats. BHB bidirectionally regulates gene expression by inducing H3K9bhb modification in rat kidneys (62). Specifically, through H3K9bhb modification, BHB extensively reprograms the chromatin structure of kidney cells, opening 3494 genomic regions and closing 7404 regions. Among them, lipid metabolism-related genes, such as Hmgcs2, Acaa1b, cytochrome P450 family 2 subfamily d polypeptide 4 (Cyp2d4), and cytochrome P450 family 2 subfamily e polypeptide 1 (Cyp2e1), are the main targets of H3K9bhb modification. The promoter regions of these genes are specifically enriched with H3K9bhb, and their mRNA and protein expression levels are significantly upregulated. In contrast to the upregulation of energy metabolism-related genes, the expression of immune inflammation-related genes (such as the Ptprc gene and Lcp1 gene encoding the CD45 protein) is significantly inhibited at both the transcriptional and translational levels, thereby alleviating renal immune damage. In terms of metabolic syndrome, researchers have reported that exogenous supplementation with BHB can induce H3K9bhb modification in multiple organs, such as the liver and kidneys, of rats (73). This modification upregulates the transcription of key lipid metabolism genes such as Hmgcs2, Cyp2d4, Cyp2e1, and Acaa1b, enhances the ability to catalyse fat decomposition, and ultimately improves the metabolic syndrome phenotype. Another study revealed that after mice fed a high-fat diet are fed a ketogenic diet, histone H3 in the liver and kidneys is modified at K4, K9, and K18; these modifications upregulate the expression of Hmgcs2 through fibroblast growth factor 21 (FGF21), promote ketone body production and enhance fatty acid oxidation, ultimately leading to weight loss, fat reduction, and blood glucose normalization in mice (74). KAT7 is a newly discovered writer enzyme for Kbhb modification. Studies have shown that the coupling of ACSS2 and KAT7 can regulate H3K9bhb modification to promote the transcription of tumour-related genes (such as SAMD9 and FRMD3), thereby affecting the proliferation and invasion of tumour cells (17). In the field of botany, Xu et al. reported that exogenous supplementation with BHB can induce H3K9bhb modification in rice and that this modification can promote the expression of defence-related genes (such as OsPR5 and OsRBOHD), thereby increasing disease resistance. Moreover, this study identified OsSRT1, OsSRT2, and OsHDA705 as eraser enzymes for Kbhb modification in rice, revealing for the first time the molecular mechanism by which H3K9bhb enhances disease resistance by regulating the expression of immune genes in plants (38). In the field of reproduction, the latest research revealed that in addition to its hypoglycaemic effect, the first-line hypoglycaemic drug metformin can upregulate the β-hydroxybutyrylation modification of histone H2B at lysine 5 (H2BK5bhb), increasing this modification in the promoter region of Gata binding protein 2 (Gata2), promoting the transcription of Gata2, and ultimately enhancing the proliferation capacity of female germline stem cells (FGSCs) (75). In the cardiovascular field, dapagliflozin can promote fatty acid oxidation and ketone body production, increasing the level of BHB in plasma and adipose tissue; BHB further promotes the expression of adiponectin through H3K9bhb modification, exerting anti-inflammatory and anti-atherosclerotic effects (76). In addition, BHB can upregulate the expression of carnitine palmitoyltransferase 1A (CPT1A) through H3K9bhb modification, promoting endothelial cell fatty acid oxidation and angiogenesis and thereby improving the cardiac repair process after myocardial infarction (77). In the neural field, BHB can induce extensive H3K9bhb modification of the brain proteome, reshaping the brain transcriptome and epigenome and ultimately affecting the expression of circadian rhythm genes and behaviour (78). Moreover, BHB can synergistically strengthen the blood–brain barrier and resist neuroinflammation through two pathways: on the one hand, it induces histone Kbhb modification; on the other hand, it promotes the enrichment of β-catenin in the promoter region of the tight junction protein zonula occludens-1 (ZO-1), jointly promoting the transcription of ZO-1 (79). In addition, BHB can activate the transcription of brain-derived neurotrophic factor (BDNF) by increasing the level of H3K9bhb in the brain, alleviating depressive behaviour in mice, and providing a new molecular mechanism for the antidepressant effect of BHB (80). Wang et al. reported that BHB-induced modification of histone Kbhb (such as H3K9bhb) can increase the transcription of mitochondrial pathway-related genes, including mitochondrial ribosomal proteins (MRPL12 and MRPS18), tricarboxylic acid cycle enzymes (CS and SUCLG1), and mitochondrial transporters (TOMM20 and TIMM9). This regulatory process can improve mitochondrial function, enhance muscle cell energy metabolism and contractile function, reverse muscle fibre atrophy, improve muscle mass and exercise capacity, and ultimately alleviate sarcopenia (81) (**Figure 5**) (**Table 3**).

**Table 3:** Research progress on histone Kbhb.

Research field	Modification sites	Inducing factor	Regulated genes	Biological effects	References
Tumor	H3K9bhb	ACSS2, KAT7	SAMD9, FRMD3	Promote the transcription of tumor-related genes and enhance the proliferation and invasion abilities of tumor cells	(17)
Kidney Disease	H3K9bhb	BHB	Hmgcs2, Acaa1b, Cyp2d4, Cyp2e1	Reduce blood pressure and protect the kidneys	(62)
Pancreatitis	H3K9bhb	BHB	Ferroptosis-inhibiting genes	Protect pancreatic cells from GSH deficiency-induced ferroptosis	(107)
Metabolic Syndrome	H3K9bhb	BHB	Hmgcs2, Acaa1b, Cyp2d4, Cyp2e1, FGF21	Promote fat decomposition	(73, 74)
Rice	H3K9bhb	BHB	OsPR5, OsRBOHD	Promote the expression of defense-related genes and enhance rice disease resistance	(38)
Germline Stem Cells	H2BK5bhb	Metformin	Gata2	Enhance the proliferation ability of FGSCs	(75)
Cardiovascular Diseases	H3K9bhb	BHB, BDH1	Adiponectin, CPT1A, LCN2, VEGF	Exhibit anti-inflammatory and anti-atherosclerotic effects; Improve cardiac repair after myocardial infarction and promote coronary revascularization; Delay DbCM; improve aortic endothelial injury in DbCM	(63, 76, 77, 98, 104)
Neurological Diseases	H3K9bhb	BHB	Circadian rhythm genes, ZO-1, BDNF	Remodel the transcriptome and affect circadian rhythms; Repair the blood-brain barrier and exert anti-neuroinflammatory effects; Alleviate depression in mice	(78–80)
Sarcopenia	H3K9bhb	BHB	MRPL12, MRPS18, CS, SUCLG1, TOMM20, TIMM9	Enhance muscle metabolism and contraction ability, reverse muscle fiber atrophy, and improve muscle mass and exercise capacity	(81)
Diabetic Nephropathy	H3K9bhb	BHB	MMP-2	Upregulate MMP-2 and counteract glomerulosclerosis in diabetic rats	(103)

## 8 Research progress on nonhistone Kbhb

To date, there has been a surge of research on histone-based Kbhb modification. Studies have shown that histone Kbhb is involved in gene transcription regulation (6), metabolic reprogramming (82, 83), and immune regulation (67, 68) and is involved in the occurrence and development of cardiovascular diseases, neuropsychiatric diseases, kidney diseases, metabolic diseases, and tumours (7). However, research in the field of nonhistone Kbhb modification remains scarce.

### 8.1 Transcriptional regulation and signal transduction

Bcl6 is a key transcription factor involved in Tfh cell differentiation and germinal centre responses; it has been shown to undergo Kac, phosphorylation, and ubiquitination, but Kbhb modification has not been previously reported. Recent studies by Guo et al. (84) first revealed that Bcl6 can undergo Kbhb modification both *in vitro* and *in vivo*. Specifically, Kbhb modification can occur at lysine residues 376, 377, and 379 of the Bcl6 protein, among which the K376 site is the main modification site. Further studies revealed that the CBP and p300 proteins are involved in the Kbhb modification of Bcl6. Functional studies revealed that after Bcl6 undergoes Kbhb modification, it can significantly inhibit the differentiation of naive CD4^+^T cells into Tfh cells, leading to a reduction in the number of Tfh cells and a simultaneous decrease in the secretion level of the cytokine IL-21. This discovery provides a new perspective and a theoretical basis for exploring the regulatory mechanism of Tfh cell differentiation and the *in vivo* immune regulation process. The p53 gene is a crucial tumour suppressor gene that is mutated and inactivated in many human cancers. Recent research has revealed that the p53 protein can undergo Kbhb modification at three main sites: lysines 319, 120, and 370. CBP/p300 can catalyse the Kbhb modification of p53; when the level of p53 Kbhb increases, it inhibits its own Kac modification, thereby downregulating the expression of downstream target genes such as p21 and PUMA (p53 upregulated modulator of apoptosis). This regulatory mechanism promotes cell proliferation and impairs the role of p53 in inducing apoptosis and regulating cell growth (85).Studies have shown that ageing promotes Kbhb modification of STAT1 at lysine 592, which inhibits the interaction between STAT1 and IFNAR2, thereby impairing IFN-I-mediated antiviral defence (61). Further studies revealed that O-GlcNAc modification of STAT1 at threonine 699 prevents CBP-induced Kbhb modification of STAT1. These findings reveal the importance of the switch between STAT1 Kbhb and O-GlcNAc modifications in regulating IFN-I antiviral immunity during ageing and provide potential strategies for improving the antiviral defence capabilities of elderly individuals. In addition, BHB can induce Kbhb modification of STAT1 at the K679 site, inhibiting LPS-induced STAT1 phosphorylation and transcription and thereby regulating the polarization of M1 macrophages (71).

### 8.2 Regulation of metabolism and energy homeostasis

In terms of metabolic proteins, AMP-activated protein kinase α2 (AMPKα2) is a key enzyme that regulates energy metabolism, protein synthesis, and glucose–lipid metabolism in cardiomyocytes. Using gene knockout technology, researchers such as Ding (86) have reported that after AMPKα2 gene deletion, some Kbhb modification sites in cardiomyocytes tend to decrease, and these sites are enriched mainly in genes related to amino acid metabolism, fatty acid metabolism, the cGMP-PKG signalling pathway, the tricarboxylic acid (TCA) cycle, and congenital heart disease-related pathways. For example, the Kbhb modification sites of carnitine O-palmitoyltransferase 1 (CPT-1)—a key rate-limiting enzyme in fatty acid β-oxidation—and pyruvate dehydrogenase (PDH) in the TCA cycle are significantly reduced after AMPKα2 gene deletion. Moreover, some Kbhb modification sites are upregulated, and the related functions involve arginine and proline metabolism, antigen processing and presentation, and the pathological process of dilated cardiomyopathy. This study is the first to elucidate the potential molecular mechanism through which AMPKα2 participates in energy metabolism regulation by regulating Kbhb modification, providing important theoretical support and research directions for the development of new therapeutic drugs targeting AMPK. HMGCS2 is a key rate-limiting enzyme in the ketogenesis process; its functional deficiency can cause mitochondrial maturation disorders in the neonatal heart, thereby hindering its normal development. Studies by the Chong team (87)revealed that the ketone body BHB regulated and produced by HMGCS2 plays a key regulatory role in postnatal heart development, promoting the maturation of cardiomyocyte mitochondria and participating in metabolic reprogramming. When ketone bodies are deficient, Kbhb modification of mitochondrial proteins is inhibited, whereas Kac modification is significantly enhanced; conversely, a moderate increase in ketone body levels in the short term after birth can effectively increase the level of Kbhb modification of mitochondrial proteins while inhibiting Kac modification. This dynamic balance of modification status is necessary for ensuring mitochondrial maturation and maintaining normal cardiac function. Notably, BHB can also improve mitochondrial function by promoting the biogenesis of mitochondria-derived vesicles (MDVs) (88). Mechanistically, sorting nexin 9 (SNX9) undergoes specific Kbhb modification, which increases the interaction between SNX9 and inner mitochondrial membrane (IMM)/matrix proteins and promotes the formation of IMM/matrix MDVs. Kbhb modification of SNX9 is not only crucial for maintaining mitochondrial homeostasis in cells but also protects mice from alcohol-induced liver injury. These findings reveal that metabolites can affect MDV formation by directly participating in key PTMs and establish a paradigm for studying how metabolites regulate mitochondrial function. Previous studies have shown that 3-oxoacid coenzyme A transferase 1 (OXCT1) is a key enzyme for extrahepatic ketone body utilization. Recent research has revealed that BHB can mediate the Kbhb of the Lye185 and 421 residues of OXCT1 to achieve self-regulation of ketone body metabolism in a negative feedback manner, thereby maintaining the homeostasis of ketone bodies in the body. Interestingly, HMGCS2 can also undergo Kbhb, but this modification does not significantly alter its enzymatic activity. This work not only helps to elucidate the mechanism underlying ketone body metabolic homeostasis regulation but also provides an important reference for the prevention and treatment of clinical conditions such as diabetic ketoacidosis (89).

### 8.3 Regulation of pathogen virulence

In terms of plant pathogenic fungi, Chen et al. (39) conducted a β-hydroxybutyrylation modification proteomic analysis of Ustilaginoidea virens and reported that in addition to histones, Kbhb modification is widely distributed on nonhistones in different cellular structures, such as the nucleus, cytoplasm, and mitochondria. KEGG analysis revealed significant enrichment in glycolysis/gluconeogenesis pathways, ribosome-related pathways, proteasome-related pathways, oxidative phosphorylation, and numerous metabolic pathways. Through protein–protein interaction (PPI) network analysis, researchers have shown that many proteins involved in the pathogenesis of plant pathogenic fungi are Kbhb-modified proteins, including MAPK pathway proteins, Septin proteins, autophagy-related proteins, and endocytosis-related proteins. Among them, Kbhb modification at K72 of mitogen-activated protein kinase (UvSlt2) and K298 of the septin protein (UvCdc10) regulates fungal virulence.This provides new insights into understanding the virulence regulation of plant pathogenic fungi.

### 8.4 Therapeutic antibodies and neurodegenerative diseases

**Figure 6:**
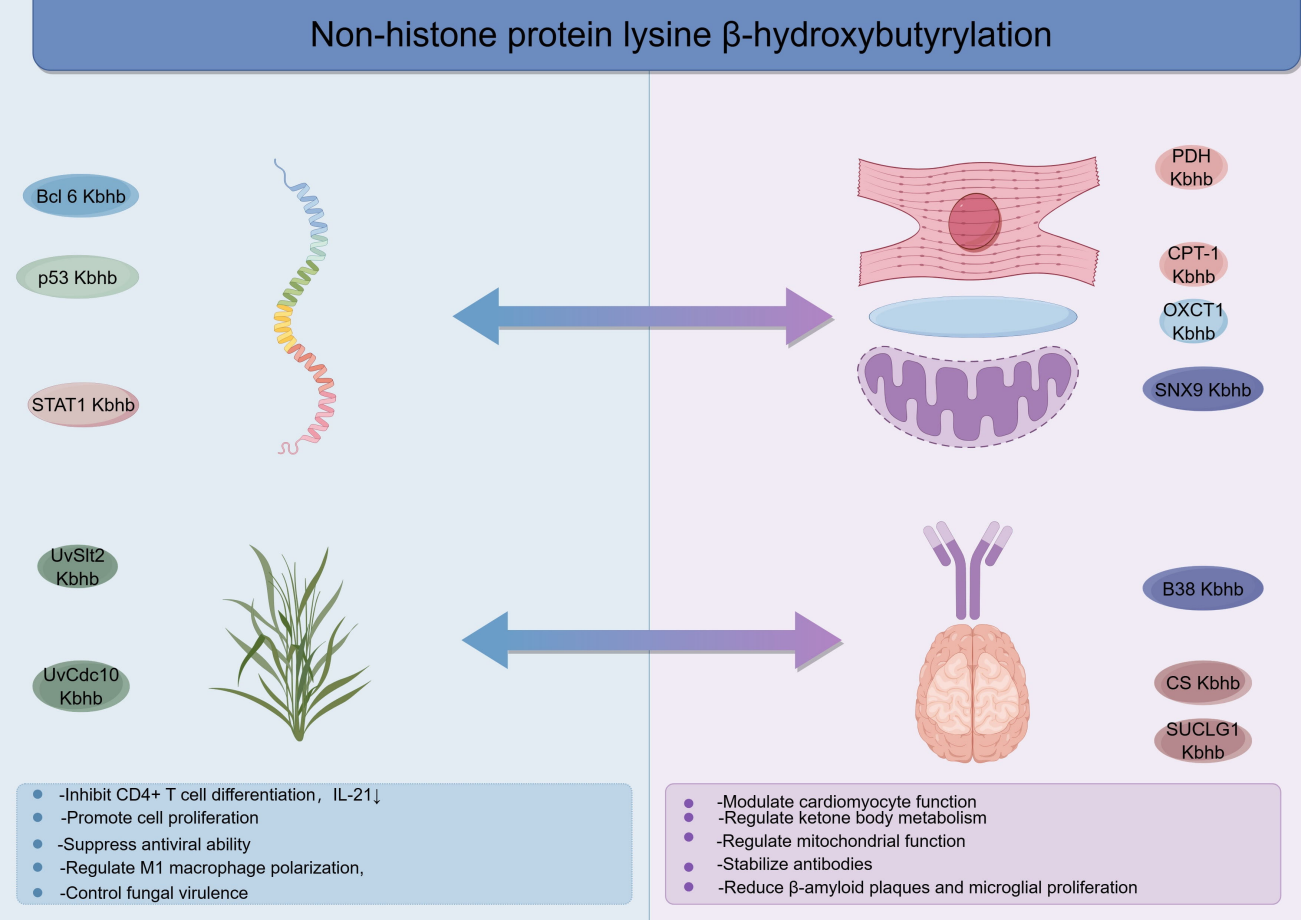
Research progress on nonhistone Kbhb.

Therapeutic antibodies have become an important class of therapeutics in the medical field because of their remarkable efficacy. Liet al. reported that the COVID-19 neutralising antibody B38, produced by 293T cells, undergoes Kbhb modification *in vivo*, which enhances the stability of the antibody. Specifically, the Kbhb modification occurs on positively charged lysine residues in the heavy and light chains of the B38 antibody (64). Alzheimer’s disease (AD) is a neurodegenerative disease characterized by extracellular plaques of β-amyloid (Aβ) and intracellular neurofibrillary tangles of tau protein and is the main cause of dementia (90). Recent research has shown that in APP/PS1 mice in the pathological stage, the levels of Kbhb modification of tricarboxylic acid (TCA) cycle-related enzymes—including citrate synthase (CS) and succinate-CoA ligase α subunit (SUCLG1)—are decreased (91). However, after a ketogenic diet is adopted, not only can the Kbhb modification level and enzymatic activity of CS and SUCLG1 significantly increase, increasing ATP production, but the pathological changes in β-amyloid plaques and microglial proliferation can also be effectively alleviated. This discovery provides a new mechanistic explanation for the beneficial effects of a KD on AD (**Figure 6**) (**Table 4**).

**Table 4:** Research progress on nonhistone Kbhb.

Research field	Proteins	Modification sites	Inducing factor	Biological effects	References
Transcriptional Regulation and Signal Transduction	Bcl6	K376, K377, K379	CBP, p300	Reduce the number of Tfh cells and decrease IL-21 secretion	(84)
Transcriptional Regulation and Signal Transduction	p53	K319, K120, K370	CBP, p300	Promote cell proliferation and weaken the functions of inducing cell apoptosis and regulating cell growth	(85)
Transcriptional Regulation and Signal Transduction	STAT1	K592, K679	Aging, CBP, LPS	Impair IFN-I-mediated antiviral defense; Regulate M1 macrophage polarization	(61, 71)
Metabolic and Energy Homeostasis Regulation	CPT-1, PDH	-	AMPKα2	Disrupt amino acid metabolism, fatty acid metabolism, and energy homeostasis in cardiac myocytes	(86)
Metabolic and Energy Homeostasis Regulation	Mitochondrial proteins	-	BHB	Maintain the dynamic balance of mitochondrial maturation and ensure postnatal cardiac development and normal cardiac function	(87)
Metabolic and Energy Homeostasis Regulation	SNX9	-	BHB	Maintain mitochondrial homeostasis and protect mice from alcohol-induced liver injury	(88)
Metabolic and Energy Homeostasis Regulation	OXCT1	Lys185, Lys421	BHB	Maintain ketone body homeostasis in the body	(89)
Pathogen Virulence Regulation	UvSlt2, UvCdc10	K72, K298	UvSirt2, UvSirt5	Regulate the virulence of Ustilaginoidea virens	(39)
Therapeutic Antibody Optimization	COVID-19 neutralizing antibody B38	Positively charged lysine residues	293T cells	Enhance the durability of antibody-mediated virus neutralization and improve the therapeutic effect on COVID-19	(64)
Neurodegenerative Disease	CS, SUCLG1	-	BHB	Increase ATP content in the brain of AD (Alzheimer’s disease) model mice and reduce β-amyloid plaque deposition and microgliosis	(91)
Specific Organ Protection	G6PDX	Lys432	BHB	Protect podocytes under high glucose stimulation	(102)
Specific Organ Protection	PHD2	Lys239, Lys385	BHB	Promote angiogenesis after myocardial ischemia and improve cardiac function	(95)

## 9 Novel therapeutic strategies

### 9.1 Metabolic regulation therapy

Currently, BHB and related Kbhb have been shown to act as cardioprotective agents, affecting gene transcription, inflammation, oxidative stress, and cardiac remodelling (92–94). In terms of promoting angiogenesis after myocardial ischaemia and protecting cardiac function, BHB binds to and induces Kbhb modification of prolyl hydroxylase domain protein 2(PHD2) at lysines 239 and 385, thereby blocking its function in the hydroxylation of hypoxia-inducible factor 1α (HIF-1α) and leading to enhanced transcription and secretion of HIF-1α-dependent vascular endothelial growth factor (VEGF), thus promoting neovascularization and cardiac repair (95). The study revealed that BHB or a ketogenic diet can be used as an adjuvant therapeutic method to promote cardiac repair, and its mechanism of action may overlap with the cardiovascular benefit mechanism of SGLT2 inhibitors; these findings also confirm that BHB can act as an endogenous PHD2 inhibitor, providing structural references and a theoretical basis for the development of new small-molecule PHD2 inhibitors. In addition, BHB can upregulate the expression of genes such as forkhead box protein O3A(FOXO3A)and MT2 to reduce oxidative stress and affect cardiomyocyte autophagy (96); during cardiac development, it can promote the maturation of mitochondria and metabolic reprogramming in cardiomyocytes (87). Studies have shown that BHB can weaken the formation of NOD-like receptor protein 3 (NLRP3) inflammasomes and antagonize proinflammatory serine-triggered mitochondrial dysfunction and fibrosis; moreover, its ability to inhibit oxidative stress and inflammation can prevent heart failure with preserved ejection fraction (HFpEF) (97). Moreover, BHB-mediated H3K9bhb modification and upregulation of carnitine palmitoyltransferase 1A (CPT1A) promote coronary revascularization, which also indicates the possibility of improving HFpEF (98). The study revealed that exogenous supplementation with BHB or a ketogenic diet can be used as a potential adjuvant therapeutic method for HFpEF. In the neural field, BHB enhances histone Kbhb modification and its interaction with β-catenin, thereby promoting the production of the tight junction protein zonula occludens-1 (ZO-1) and alleviating lipopolysaccharide (LPS)-induced excessive blood–brain barrier permeability (79). The results of the study provide a new potential target—the Kbhb–β-catenin–ZO-1 axis—for the clinical treatment of neuroinflammation-related CNS diseases such as Alzheimer’s disease and stroke. Future development of drugs targeting this pathway, such as new BHB supplements or combinations with Wnt pathway modulators, is expected to yield new therapeutic strategies. Notably, after BHB is taken up by monocarboxylate transporter 1 (MCT1), which is specifically expressed in brain endothelial cells, it can upregulate the expression of ZO-1 by enhancing the local β-hydroxybutyrylation of H3K9 at the TJP1 gene promoter, promoting the repair of the blood–brain barrier after ischaemic stroke (99). The results of the study reveal a new mechanism by which BHB protects the integrity of the blood–brain barrier through the H3K9bhb–ZO-1 axis after stroke, and exogenous supplementation with BHB or a ketogenic diet may become a potential neuroprotective adjuvant therapeutic strategy. In terms of the decline in the body’s antiviral ability caused by ageing, STAT1 undergoes two novel modifications: Kbhb modification and O-GlcNAc modification. These two modifications regulate IFN-I signalling through antagonistic effects, thereby affecting the antiviral ability of elderly individuals (61). Studies have shown that the anticancer drug hydroxycamptothecin can effectively reduce the level of STAT1 Kbhb, thereby enhancing the IFN-I signalling and antiviral defence ability in elderly mice. Fructose supplementation can increase the O-GlcNAc modification of STAT1, thereby inhibiting Kbhb modification and reshaping the antiviral immune response ability of aged mice (61). These findings suggest the possibility of clinically targeting the modification status of STAT1 through drugs (hydroxycamptothecin) or dietary/metabolic interventions (fructose) to improve the antiviral immunity of elderly individuals. In CRPC, OTUD7B can undergo Kbhb modification, which inhibits the cGAS-STING signalling pathway and reduces interferon expression to impair the body’s immune response ability. These findings suggest that the BHB–OTUD7B–Kbhb axis may become a new target for reversing immune resistance (45). Therefore, clinically, inhibitors targeting OTUD7B Kbhb modification can be developed to restore the function of the cGAS-STING pathway, promote the production of type I interferons, and thus restore the antitumour function of the tumour immune microenvironment. ALDOB Lys108bhb can downregulate mTOR signalling and glycolysis levels by reducing its binding ability to FBP, significantly inhibiting the proliferation of various tumour cells, such as renal, gastric, and liver cancer cells (23). Therefore, drugs that directly simulate the effects of ALDOB K108bhb or target key nodes of the mTOR pathway can exert antitumour effects. Third-generation ATP-competitive mTOR inhibitors (such as BI 860585) can simultaneously inhibit mTORC1/2 and overcome resistance to first-generation drugs; however, they are still in the clinical research stage (100). Notably, in LUAD, BDH1 inhibitors (such as pimozide and crizotinib) can synergistically inhibit the proliferation of LUAD cells with high BDH1 expression by blocking H3K9bhb modification (55).

### 9.2 Targeted therapy

Recent research has shown that resveratrol (RES) can inhibit hypoxia-induced nerve damage by inhibiting the production of reactive oxygen species (ROS), increasing the level of Kbhb, and reducing inflammation. The discovery of this molecule provides new ideas for the diagnosis and treatment of stroke and can be used as a new target for the clinical treatment of stroke (101). Hu et al. reported that BHB can induce β-hydroxybutyrylation modification of 6-phosphogluconate dehydrogenase (G6PDX) at lysine 432, promoting the expression and activity of G6PDX and thereby protecting podocytes under high-glucose stimulation (102). In addition, BHB treatment can upregulate the production of matrix metalloproteinase-2 (MMP-2) by increasing the content of H3K9bhb in the MMP-2 promoter, thereby counteracting glomerulosclerosis in diabetic rats (103). Therefore, BHB is expected to become a new target for the treatment of diabetic nephropathy.In diabetic cardiomyopathy (DbCM), treatment with the histone β-hydroxybutyrylation inhibitor A485 delays the progression of DbCM, and BDH1 can be used as a new target for DbCM treatment (63). Studies have shown that overexpression of BDH1 decreases the level of BHB and H3K9bhb modification in the promoter region of lipocalin 2 (LCN2) and inhibits DbCM by inhibiting LCN2-dependent NF-κB activation. Clinically, the H3K9bhb inhibitor A485 can be used to reduce LCN2 expression; at the same time, monoclonal antibodies targeting LCN2 can be developed to bind and clear LCN2 protein in the circulation, preventing its interaction with NF-κB/RPS3; alternatively, small-molecule compounds that can block the binding of LCN2 to the RPS3/NF-κB complex can be explored. Notably, Wu et al. demonstrated that exogenous supplementation of BHB in streptozotocin (STZ)-induced SD rats with type 2 diabetes upregulates the level of histone H3K9bhb modification in the rat heart in a dose-dependent manner, leading to the expression of vascular endothelial growth factor (VEGF) and subsequently improving aortic endothelial damage in the heart in DbCM (104). This study provides a solid theoretical foundation for the development of BHB as a new therapeutic molecule for the prevention and treatment of diabetic vascular complications such as atherosclerosis and hypertension. Studies have shown that metabolomics is a new therapeutic strategy centred on mitochondrial oxidative metabolism; targeting Kbhb sites on mitochondrial proteins provides a potential therapeutic approach for patients with high-risk cancer (105). MRPL13 is a gene related to immune infiltration and tumour microenvironment remodelling; the latest research revealed that mitochondrial ribosomal protein L13 (MRPL13) is highly expressed and undergoes Kbhb modification in gastric cancer tissues, indicating that MRPL13 can be used as a potential therapeutic target for gastric cancer (106). MRPL13 knockdown can upregulate the expression of pro-apoptotic genes (BAX and cleaved caspase-3) and downregulate the expression of anti-apoptotic genes (BCL-2) to significantly increase apoptosis; it also inhibits the expression of epithelial–mesenchymal transition (EMT) markers and increases the expression of epithelial markers such as E-cadherin to reduce migration and invasion potential. Small-molecule inhibitors or RNA-based therapies targeting MRPL13 can be used as new therapeutic strategies. Previous studies have shown that Kbhb modification of p53 is related to cancer progression, suggesting that targeting p53 Kbhb may be a feasible therapeutic method (85). Targeting this new mechanism of p53-Kbhb provides a theoretical basis for the development of combined tumour therapeutic strategies involving the Kbhb enzyme (such as CBP/p300) inhibitor A485. In addition, studies have reported that targeting Kbhb sites on CD8^+^T-cell proteins can increase the expression of CPS1; this ammonia-scavenging mechanism may be used to improve T-cell-based cancer immunotherapy (68). Moreover, this study provides a strong theoretical basis and potential targets for the clinical use of stable analogues of allosteric CPS1 activators (such as N-carboxyglutamic acid (NCG)) or BHB to increase the efficacy of T-cell antitumour immunotherapy. Zheng et al. (107) reported that ferroptosis caused by glutathione (GSH) deficiency is the cause of pancreatic injury in mice with acute liver failure (ALF). Studies have shown that BHB can protect pancreatic cells and tissues from ferroptosis caused by GSH deficiency. The administration of BHB to ALF mice can restore the expression of ferroptosis-inhibiting genes through H3K9bhb-mediated chromatin opening. These findings reveal the important mechanism through which BHB acts as an endogenous metabolite in the regulation of pancreatic ferroptosis, provide a new drug intervention target for the treatment of acute liver failure complicated with pancreatitis, and reveal a new path for improving the clinical treatment effect of patients with acute liver failure.

## 10 Conclusion and prospects

In recent years, research on Kbhb modification has increased significantly. Studies have confirmed that it has a wide range of functions; participates in processes such as energy metabolism, tumorigenesis, and DNA damage repair (30); and plays key roles in KD regulation, tumour progression, immune regulation, and disease development, with a close connection to ketone body metabolism. The biological effects of the ketogenic diet mediated by ketogenic metabolism may be the core “code” for interpreting the mechanism of KD. Notably, KD has shown potential in the treatment of various cancers (23). However, its mechanism of action remains unclear; the specific pathway by which KD regulates tumour cell metabolism and energy supply through enhancing Kbhb modification can be explored from the perspective of metabolic reprogramming, and in-depth analysis of the Kbhb regulatory mechanism of key ketone body metabolism enzymes such as BDH1 and HMGCS2 can also provide an important basis for clarifying the pathological mechanism of diseases. These findings suggest that in the future, a KD can be used to increase the level of BHB in the body and regulate the Kbhb modification of key molecules to delay disease progression; however, the long-term safety of this strategy (such as the risk of ketoacidosis) and individualized dosage specifications still need to be verified in clinical studies. In addition, the development of Kbhb modification site prediction technology (25), the clarification of the substrate specificity of Kbhb “writers” (such as p300/CBP) and “erasers” (HDACs/SIRTs) (30), and the development of related targeted inhibitors/activators are important research directions in the current field. At present, shortcomings in Kbhb research still exist. First, the specific regulatory pathway of Kbhb in cancer occurrence and development has not been clarified, and research on the microenvironment dependence of procancer and anticancer effects (such as differences in the expression of BDH1 or ketone body-utilizing enzymes in different tumour subtypes) lacks systematic analysis. Second, as a new posttranslational modification, research on Kbhb “readers” is extremely scarce, and the exploration of related regulatory elements is also obviously insufficient. Third, the interaction between Kbhb and other acylations, such as Kac and succinylation, has not been clarified. Fourth, research on nonhistone Kbhb modification is relatively weak. Although its role in regulating pathogenic mechanisms has been identified in plant pathogenic fungi and has shown potential in T-cell memory formation, macrophage polarization, and mitochondrial function regulation, the depth and breadth of overall research still need to be expanded. Therefore, future research should focus on the following topics: prioritizing the identification of Kbhb “readers” and exploring regulatory elements; clarifying the substrate specificity of the Kbhb “write–read–erase” system; laying a molecular foundation for subsequent targeted drug development; analysing the microenvironment dependence of Kbhb in cancer; establishing tumour subtype-specific intervention strategies; clarifying the anticancer mechanism of KD combined with Kbhb modification from the perspective of metabolic reprogramming; focusing on related targets and signalling pathways; exploring the disease treatment potential of exogenous BHB supplementation; focusing on how BHB affects cellular stress responses and cell death pathways through Kbhb modification, thereby improving the pathological state of diseases; strengthening research on nonhistone Kbhb modification; expanding its translational application in immunometabolism, pathogen prevention and control; and clarifying the interaction network between Kbhb and other acylations, ultimately promoting the transformation of Kbhb basic research to clinical diagnosis and treatment.
